# Citrus Peel Extracts: Effective Inhibitors of Heterocyclic Amines and Advanced Glycation End Products in Grilled Pork Meat Patties

**DOI:** 10.3390/foods13010114

**Published:** 2023-12-28

**Authors:** Yang Xu, Guangyu Li, Lan Mo, Maiquan Li, Jie Luo, Qingwu Shen, Wei Quan

**Affiliations:** 1 College of Food Science and Technology, Hunan Agricultural University, Changsha 410128, Chinayaoyao3153@aliyun.com (Q.S.); 2 State Key Laboratory of Food Science and Technology, Jiangnan University, Wuxi 214122, China

**Keywords:** citrus peel, polymethoxylated flavonoids, heterocyclic amines, advanced glycation end products, pork meat patties

## Abstract

In the present study, citrus peels were extracted using various conventional and deep eutectic solvents (DESs). Compared to other citrus peel extracts, the DES extract based on choline chloride showed notably higher total phenolic and flavonoid content levels, along with superior antioxidant activity, among these extracts. Consequently, this study aimed to further investigate the inhibitory effects of the choline chloride based DES extract on the production of both free and bound heterocyclic amines (HAs) and advanced glycation end products (AGEs) in roast pork meat patties. The results indicated that the addition of choline chloride-based DES extracts, particularly the choline chloride-carbamide based DES extract, can effectively reduce the oxidation of lipids and proteins by quenching free radicals. This approach proves to be the most efficient in reducing the formation of both HAs and AGEs, leading to a significant reduction of 19.1–68.3% and 11.5–66.5% in free and protein-bound HAs, respectively. Moreover, the levels of free and protein-bound AGEs were reduced by 50.8–50.8% and 30.5–39.8%, respectively, compared to the control group. Furthermore, the major phenolics of citrus peel extract identified by UHPLC-MS were polymethoxylated flavonoids (PMFs) including hesperidin, isosinensetin, sinensetin, tetramethoxyflavone, tangeretin, and hexamethoxyflavone, which inferring that these compounds may be the main active ingredients responsible for the antioxidant activity and inhibition effects on the formation of HAs and AGEs. Further research is needed to explore the inhibitory effects and mechanisms of PMFs with different chemical structures on the formation of HAs and AGEs.

## 1. Introduction

Inhibiting the formation of a series of harmful compounds during high-temperature food processing has become a significant topic within the field of food safety research [1,2,3]. While high-temperature processing can enhance the flavor, color, and texture of food, it can also result in adverse consequences, particularly the formation of harmful compounds like heterocyclic amines (HAs) and advanced glycation end products (AGEs) [2,4,5,6]. Previous studies have confirmed that HAs are a class of mutagenic and carcinogenic substances which formed in cooked meats as a result of the Maillard reaction. So far, a variety of over 30 different HAs has been discovered in different foods. Certain HAs have been categorized by the IARC as human carcinogens of Class 2A and 2B. It is recommended to limit the consumption of these compounds for the sake of one’s health [4,7]. Furthermore, AGEs are detrimental chemicals that arise from nonenzymatic reactions, including polymerization and condensation reactions. Researchers have substantiated that the overabundance of endogenous AGEs obtained from daily dietary intake can be amassed within human bodies after digestion and absorption. This accumulation subsequently leads to adverse consequences, like heightened oxidative or inflammation stress and an elevated risk of various ailments [8,9,10].

Given the possible health risks associated with the formation of HAs and AGEs, researchers have explored several potential strategies to inhibit their formation in various food systems. Among these strategies, the addition of exogenous antioxidants is widely considered as one of the most effective means to inhibit the generation of HAAs and AGEs [11,12,13,14]. Research shows that natural antioxidants such as herbs, vegetable or fruit extracts can effectively inhibit the formation of AGEs or HAs through several pathways such as scavenging free radicals, capturing reactive carbonyl intermediate compounds, etc. [11,15,16,17]. However, these natural antioxidants are prone to oxidation and degradation reactions during high-temperature processing, which not only significantly reduces their antioxidant activity but also poses safety risks [18,19]. In addition, research has also found that antioxidants have adverse effects on the quality of thermal processed food, and their industrial use is costly [18,19]. All of these factors limit the application of antioxidants in the processing of meat products. Thus, to enhance the suppressive impact of indigenous antioxidants on HAs and AGEs in thermally processed foods, scientists have directed their attention towards extracts of natural products that possess a wider range of origins, superior thermal stability, and heightened antioxidant efficacy.

Citrus fruits are among the most abundant fruits in the world. The annual production of citrus fruits exceeds 124 million tons worldwide, and the citrus fruit and fruit juice industry has seen continuous growth [20,21]. In recent decades, approximately one-third of citrus fruits have been processed, resulting in a significant amount of waste each year. Citrus peels, which make up a substantial portion of the total fruit weight, remain as the predominant residue [21]. Citrus peels possess substantial quantities of biologically active polyphenols, particularly polymethoxylated flavonoids, which have demonstrated vital antioxidant characteristics [22,23]. In Eastern Asian regions, fresh citrus peels experienced dehydration and treatment to serve as a functional nutritional component [21]. Consequently, waste derived from citrus peel in the juice industry could potentially be perceived as an invaluable and secure reservoir of bioactive substances contributing to health enhancement, such as phenolic compounds, with potential interest as a natural antioxidant additive in the application of inhibiting harmful substances such as HAs and AGEs.

However, there are limited studies focusing on the inhibited effect of citrus peel extract on the formation of HAs and AGEs. Therefore, in this study, citrus peel extracts were prepared using both traditional solvents and green deep eutectic solvents (DES), and their antioxidant activity and the composition of phenolic compounds were analyzed. Moreover, the inhibition effects of citrus peel extract on the formation of HAs and AGEs in grilled pork meat patties were also determined.

## 2. Materials and Methods

### 2.1. Reagents and Chemicals

Citrus peel from orange (*Citrus sinensis*) and Longissimus thoracis (LT) muscle from pig (Duroc × Landrace × Yorkshire, DLY) were purchased from a supermarket in Changsha City. Toronto Research Chemicals Inc. provided a total of 17 HA and 2 AGE standards. Waters (Milford, MA, USA) was the source of Oasis MCX SPE column. Choline chloride, L-carnitine, betaine, acetamide, ethylene glycol, carbamide, and additional analytical grade chemicals were purchased from Sinopharm Chemical Reagent Co., Ltd. (Shanghai, China).

### 2.2. Preparation of Citrus Peel Extracts

Preparation of citrus peel extracts by conventional extraction solvents: Citrus peel powders (5.0 g) were prepared via ultrasonicate-assisted extraction with distilled water, ethanol, or 80% ethanol solutions (*w*/*v* = 1:25) at 45 °C, 40 kHz, and 480 W for 1 h, and the above solution was centrifuged (10,000× *g*, 10 min) to obtain the supernatant. Following three rounds of extraction and centrifugation, the resulting supernatants were collected and named as water, EtOH, and 80EtOH.

Preparation of citrus peel extracts by DES [24,25]: As shown in Table 1, transparent solutions were obtained by mixing choline chloride, L-carnitine, and betaine solutions in a 1:2 molar ratio with hydrogen bond acceptors, followed by stirring at 300 rpm and heating in a water bath at 75 °C. Next, generated DES were diluted with distilled water to attain a concentration of 75%. Additionally, the citrus peel powder was mixed with the 75% DES at a solid to solvent ratio of 1:25 and then also treated via ultrasonicate-assisted extraction with a KQ-300E ultrasonic machine (Kunshan Ultrasonic instruments Co., Ltd., Kunshan, China) at 45 °C, 40 kHz, and 480 W for 1 h. Similarly, after three rounds of extraction and centrifugation, the resulting supernatants were collected and named as DES1-DES9.

### 2.3. Determination of Total Phenolic Content (TPC)

As previous published literature [11], 100 μL diluted sample was combined with 100 μL of Folin–Ciocalteu reagent and left to mix for a duration of 3 min. After that, a solution containing 800 μL of Na_2_CO_3_ (75%) was added to the mixture. The mixture was then stored in a dark environment at 25 °C for a period of 2 h. To determine the concentration, the absorbance of the reacted solution was measured at a wavelength of 765 nm. A standard curve was created using gallic acid, and the final result was expressed as mg GAE/g DW.

### 2.4. Determination of Total Flavonoid Content (TFC)

The modified Davis spectrophotometric method was conducted to determine the TFC of citrus peel extracts [26,27]: 1 mL extract was mixed with 4 mL distilled water, diethylene glycol (90%, 5 mL), and 4 M NaOH (0.1 mL) separately. To measure the absorbance, the samples were placed in a water bath for 10 min at a temperature of 40 °C. After this, the samples were allowed to cool down to room temperature. The absorbance was then measured at two different wavelengths, 360 nm and 420 nm. In order to establish a reference, standard compounds hesperidin and naringin were used at the respective wavelengths of 360 nm and 420 nm. The concentration range for hesperidin was 0–400 µg/mL, while for naringin, it was 0–250 µg/mL.

### 2.5. Antioxidant Activity of Citrus Peel Extracts

According to Quan et al. [28,29], a mixture of 10 µL sample and 190 µL of the ABTS radical cations (ABTS*+) working solution was combined and incubated at 25 °C for 10 min. Following incubation, the absorbance of the solution undergoing the chemical reaction was measured at a wavelength of 734 nm. The FRAP value was calculated as previously reported by Qie et al. [30]: In the experiment, 10 µL extract solutions were mixed with 190 µL FRAP working solution. After incubating for 30 min, the absorbance of the mixture was measured at 593 nm. Antioxidant capacity of the samples was determined by comparing them to a standard of Trolox.

### 2.6. Pork Meat Preparation and Cooking

Ground pork meat was supplemented with 1% citrus peel extract derived from DES. To ensure consistency, the pork meat was then shaped into patties using a Petri dish (Φ 6 cm × 1.5 cm), with each patty containing approximately 40 ± 0.1 g of raw meat [11,15]. The patties were subsequently cooked in a Cooking Center oven (Landsberg, Munich, Germany) at a temperature of 225 °C for 10 min on each side. Following cooling to room temperature, the cooked patties were stored at −18 °C until they were ready for analysis.

### 2.7. Qualitative and Quantitative Extraction and Determination of HAs

For the extraction of free HAs, 40 mL 3M NaOH solution, 2 g grilled pork meat samples and 20 mL ethyl acetate was added to the solution, and the sample was processed with ultrasound (40 kHz, 40 min). The above solution was centrifuged (3000× *g*, 10 min) to obtain the supernatant, and solid phase extraction of the supernatant was conducted referred to previous reported method [11,31].

For the extraction of protein-bound HAs, the precipitate after centrifugation mentioned above was thoroughly mixed with an 20 mL 6M hydrochloric acid solution and then reacted at 110 °C for 24 h. Finally, the mixed solution was filtered and diluted to 100 mL before solid-phase extraction.

For the measurement of both free and protein bound HAs, Waters UPLC-QQQ-MS (Waters, USA) equipped with BEH C18 inverted column (2.1 mm × 100 mm, 1.7 μm) was selected. The configuration of a binary mobile phase consisting of acetonitrile (A) and 0.1% formic acid (B) was necessary for the gradient elution process. The procedure involved the following steps: 0–0.1 min 4% A; 0.1–12 min, 4–20% A; 12–14 min, 20–100% A; 14–17 min, 100–4% A. For the detection process using mass spectrometry, multiple reaction monitoring scanning settings were employed. The specific parameter settings were as follows: the capillary voltage was adjusted to 3.5 kV, the ion source temperature was set to 120 °C, the specified temperature for solvent removal was 350 °C, the flow rate of the nitrogen gas for the conical gas flow was set at 60 L/h, and the desolvent gas (nitrogen) had a flow rate of 650 L/h.

### 2.8. Determination of AGEs

For the extraction of protein bound AGEs [11,32], firstly, 30 mg sample and 3 mL of n-hexane were centrifuged for 15 min. The sample was next reduced with 1.5 mL sodium borate buffer and 1 mL sodium borohydride at 4 °C for 12 h, then 2.5 mL HCl was added to the mixed solution. Finally, the solution was heated to 110 °C to facilitate hydrolysis. The hydrolysate was diluted to 10 mL. SPE was conducted on 2 mL of the redissolved hydrolysates subsequent to filtration. 

The analysis of AGEs was conducted using UPLC-QQQ-MS. According to previous literature, a UPLC system was used to inject 5 μL of samples, which were then separated in a T3 column (150 × 2.1 mm, 3.5 μm) maintained at 35℃. The gradient elution conditions were as follows: 1% A for 0–0.1 min, 1–3% A for 0.1–3 min, 3–100% A for 3–7 min, 100% A for 7–9 min, and 100–1% A for 9–10 min. The flow rate was set at 0.2 mL/min. And the mass spectrometric data was acquired using ESI+ mode and MRM.

### 2.9. Protein and Lipid Oxidation of the Grilled Meat Patties

Lipid and protein oxidation of the grilled pork meat patties were determined by measuring POV, TBARS, and total carbonyl content using a commercial kit [11,33].

### 2.10. Identification of Free Radicals in Fried Meatballs 

In order to determine the content of free radicals in the sample, 0.1 g grilled pork meat sample was added to NMR tube. Parameters for ESR analysis was set as follows: central field, 3360 G; scanning width, 100 G; microwave power, 20 mW; modulation amplitude, 1.0 G; and scanning time, 60 s [31].

### 2.11. Quality Analysis of Grilled Meat Patties

Protein, ash, moisture contents, pH value, and cooking loss of grilled pork meat samples were analyzed according to AOAC procedures [34].

Texture profile analysis was conducted by TA-XT plus texture analyzer (Godalming, UK). The probe for texture profile analysis was a P/50 cylinder probe, and the parameters of texture analyzer were as in previous studies [11].

### 2.12. Identification of Phenolic Compounds Using UPLC-PDA-QTOF-MS

The Waters UPLC equipped with 2996 DAD detector was initially used to separate citrus peel extracts. The elution program involved a gradient with the following settings: 0–2 min at 2% A, 2–20 min at 2–30% A, 20–24 min at 30–80% A, 24–26 min at 80–100% A, and 26–30 min at 100–2% A. Mobile phase A in this program was made up of 0.1% formic acid and water, while acetonitrile composed mobile phase B. The column temperature remained at 35 °C, and the detector wavelengths were set at 280 nm. 

### 2.13. Statistical Analysis

The experimental results were based on the average standard deviation, and a variance analysis was conducted to identify significant differences among the treatment groups. All samples were subjected to three separate and independent experiments. A *p* value < 0.05 was used to establish statistical significance.

## 3. Results and Discussion

### 3.1. TPC and TFC of Citrus Peel Extracts

**Figure 1:**
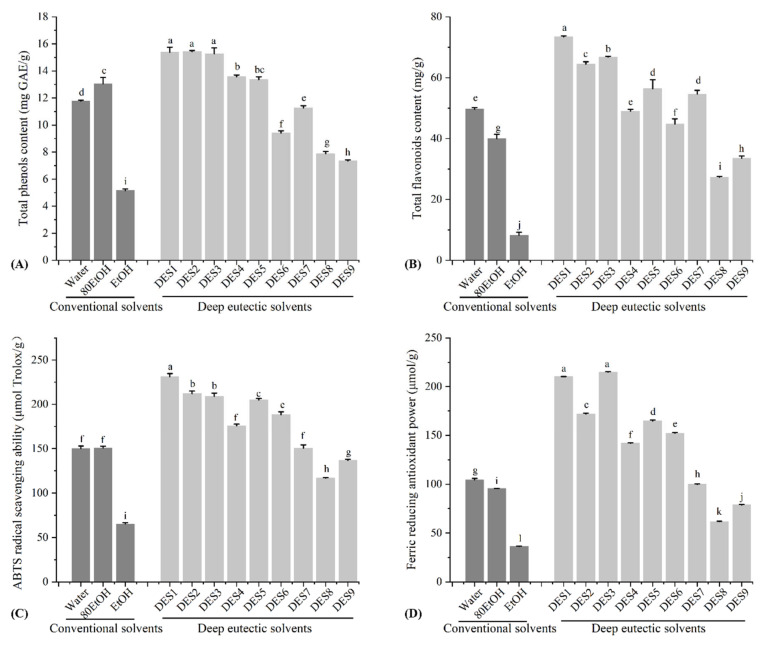
Antioxidant capacity and phenolic contents of different citrus peel extracts. (**A**) Total phenolic contents, (**B**) total flavonoids contents, (**C**) ABTS*+ radical scavenging ability, (**D**) ferric reducing antioxidant power. Different lowercase letters denote significant differences (*p* < 0.05).

According to previous studies, citrus peel is rich in polyphenols and flavonoids [21,23]. As shown in Figure 1A, TPC in the citrus peel extract obtained using a traditional solvent ranged from 5.15 to 13.04 mg GAE/g. Among the different extraction methods, citrus peel extracted using 80% ethanol exhibited the highest value of TPC. While citrus peel extracted by ethanol showed the lowest TPC value. However, when compared to conventional solvents, the TPC of the extracts from citrus peels using DES was significantly higher, except for DES 7, 8, and 9 which were based on L-carnitine. Choline chloride based DES demonstrated the highest TPC, reaching 15.2–15.4 mg GAE/g, followed by DES 4 and 5. Moreover, citrus peel extracts showed similar TPC with that of TPC. The TFC of citrus peel extract obtained from ethanol was the lowest among the solvents tested, reaching only 8.21 ± 0.96 mg/g (Figure 1B). Despite being significantly higher than the other two conventional solvents, the TFC of the water extract still remained considerably lower than that of DES, particularly DES1, 2, 3, 5, and 7, which ranged from 54.6 ± 1.25 to 73.4 ± 0.34 mg/g. In general, when compared to different DES, choline chloride-based DES, such as DES1, DES2, and DES3, demonstrated significantly higher TPC and TFC than other DES. Notably, DES1 exhibited particularly high TPC and TFC compared to the rest. On the other hand, DES based on L-carnitine, especially DES7, 8, and 9, has shown TPC and TFC values comparable to or less than 80% EtOH and water. However, their TPC and TFC were significantly lower than those of other DES. 

### 3.2. Antioxidant Activities of Citrus Peel Extract

The antioxidant activity of citrus peel extract with nine kinds of DESs and three kinds of conventional solvents was evaluated using ABTS*+ and FRAP antioxidant assays. In the antioxidant assays, it is observed in Figure 1C,D that the citrus peel extract from ethanol had significantly lower free radical scavenging activities compared to the other solvents (*p* < 0.05). This finding is consistent with previous results, which indicated that the citrus peel extract from ethanol had the lowest levels of TPC and TFC. DES1, 2, 3, 5, and 6 showed significantly higher ABTS*+ scavenging activities compared to conventional solvents. The DES based on choline chloride displayed the highest ABTS*+ radical scavenging activities out of all these options, with betaine-based DES coming in next. The FRAP values of citrus peel extract show a parallel pattern to the ABTS*+ scavenging activities of citrus peel obtained from different solvents. These findings also demonstrate the excellent antioxidant activity of both choline chloride and betaine-based DES. However, it is worth noting that the antioxidant activities of L-carnitine-based DES are significantly lower than that of water and 80EtOH. The respective range of DES7, 8, and 9 in the two antioxidant assays were only 116.8 ± 0.52 to 150.6 ± 3.66 μmol Trolox/g in the TEAC assay and 61.6 ± 0.39 to 99.8 ± 0.59 μmol Trolox/g in the FRAP assay. Similar results have shown that DES extracts have better antioxidant activity compared to those obtained using conventional solvents. Recent findings suggest that certain components of DES can enhance the antioxidant activity of the extracts. This suggests that there might be a synergistic effect between DES and soluble compounds [25,35].

### 3.3. Effects of Citrus Peel Extract on the Production of HAs in Meat Patties

Considering that choline chloride-based DES extract exhibits the strongest antioxidant activity among all citrus peel extracts, this study chose to investigate the inhibitory effects of these types of extracts on HAs and AGEs in grilled pork meat. The effects of citrus peel extract on the production of free and bound HAs in the pork meat patties were determined and are shown in Table 2. 

In the control group, six kinds of free HAs and eight kinds of protein bound HAs were detected, with their total contents reaching 14.01 ng/g and 973.4 ng/g, respectively, which is consistent with the results reported in previous studies [17]. Furthermore, all three choline chloride-based DES extracts significantly reduced the overall levels of free and protein bound HAs in pork meat patties to a range of 34.3–37.1% and 33.2–41.8%, respectively. Among these extracts, DES3 displayed the strongest inhibitory effect on the formation of HAs. In detail, apart from harmane, the citrus peel extracted from choline chloride-based DES significantly reduced the formation of free PhIP, MeIQx, 7,8-DiMeIQx, AαC, and norharmane. Specifically, the levels of PhIP, MeIQx, and AαC were significantly decreased by 49.2–68.3%, 34.7–53.2%, and 56.6–77.4%, respectively. Similarly, the choline chloride-based citrus peel extract also exhibited a strong inhibitory effect on the formation of bound forms of PhIP, MeIQx, 7,8-DiMeIQx, AαC, and norharmane, with reductions of 48.0–63.1%, 43.6–52.1%, 29.2–58.9%, 54.9–66.5%, and 50.4–58.9%, respectively. However, the inhibitory effect of citrus peel extract is limited for the other three kinds of protein-bound HAs, namely 1,5,6-TMIP, Harmane, and MeAαC. This result suggests that citrus peel extract has the ability to inhibit both free and protein-bound HAs, particularly AIAs HAs.

### 3.4. Effects of Citrus Peel Extract on the Formation of AGEs in Pork Meat Patties

As shown in Table 3, the control group showed detectable levels of two types of AGEs, CML and CEL, which mainly exist in both free and bound forms. Among these, the free AGE content was found to be 147.6 μg/g, while the protein-bound AGE content was 7.39 μg/g, which aligns with previously reported findings in the literature [11,16]. Obviously, the citrus peel extract showed an inhibitory effect on free CML and CEL, reducing them by 40.1–50.8% and 41.8–52.2%, respectively. However, the citrus peel extract showed a lower inhibitory effect on the production of protein-bound CML and CEL, only reducing them by 17.1–39.8% and 16.8–30.5%, respectively. In comparison with those three choline chloride-based DES extracts, DES3 exhibited a better inhibitory capacity, which significantly decreased the content of both free and protein-bound CML and CEL. These findings are consistent with the effects observed from the use of citrus peel extract on the formation of HAs, as reported earlier.

### 3.5. Effects of Citrus Peel Extract on Protein and Lipid Peroxidation

During thermal processing, lipids and proteins tend to undergo oxidative degradation, resulting in the creation of reactive carbonyl compounds. These compounds can further increase the buildup of detrimental AGEs and HAs [2,36,37]. It has been previously shown in research that specific plant extracts and their bioactive compounds can potentially hinder the production of AGEs and HAs. This is achieved by either removing free radicals or reducing the concentrations of active carbonyl intermediates in the system [14,37]. As mentioned earlier, citrus peel extracts have shown excellent antioxidant activity. Hence, the primary objective of this study is to reinforce this hypothesis by examining alterations in free radicals and the concentration of oxidation products within pork meat patties.

**Figure 2:**
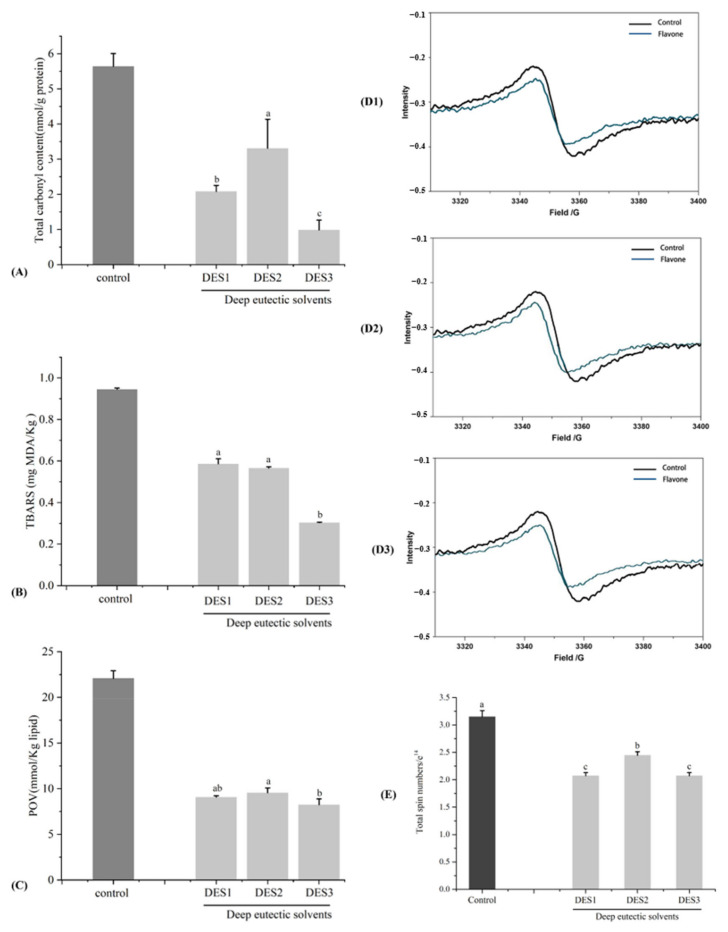
Citrus peel extract on free radicals, lipid oxidation, and protein oxidation of meat patties. (**A**) Total carbonyl content, (**B**) TBARS value, (**C**) POV value, (**D1**–**D3**) the ESR spectrum of free radicals in grilled meat patties add with DES1–DES3, (**E**) the total spin number of grilled meat patties. Different lowercase letters denote significant differences (*p* < 0.05).

Protein carbonyls, produced during the oxidative breakdown of proteins, indicate the extent of protein oxidation. To determine the degree of lipid oxidation, TBARS and POV values are commonly used [33]. As shown in Figure 2, the control group had a total carbonyl content of 5.64 ± 0.36 mmol/kg, TBARS of 0.95 ± 0.01 mg/kg, and POV values of 22.1 ± 0.84 mmol/kg. These values were slightly lower than those reported in the literature [16,17]. The extracts of choline chloride-based DES significantly reduced the content of total carbonyl to 0.98–3.31 mmol/kg, TBARS, and POV values in pork meat patties to 0.48–0.49 mg/kg, and 8.86–9.05 mg/kg, respectively. DES1 and DES2 exhibited comparable impacts on protein and lipid peroxidation among the DES extracts. Nonetheless, DES3 effectively diminished the oxidative deterioration of lipids and proteins in roasted pork meat patties. This aligns with DES3’s ability to decrease the content of HAs and AGEs.

Electron paramagnetic resonance (ESR) is an effective method for detecting free radicals [38]. The ESR spectrum of free radicals in grilled pork meat patties, with the addition of choline chloride-based DES extracts, is shown in Figure 2D. Compared to the control group, the amplitude of the detection spectrogram for the grilled pork meat patties enriched with choline chloride-based DES extracts showed a decreasing trend, which was most pronounced in the samples enriched with DES1 and 3. Figure 2E shows the total spin number of the experimental group of grilled pork meat patties that were added with choline chloride-based DES extracts. In comparison to a spin number of 3.18 ± 0.04 × 10^14^ in the control group, the spin numbers in DES1, 2, and 3 were significantly decreased to 2.05 ± 0.03 × 10^14^, 2.51 ± 0.05 × 10^14^, and 2.10 ± 0.03 × 10^14^, respectively. In general, the outcomes of the present research suggest that extracts containing choline chloride-based DES have the potential to diminish lipid and protein peroxidation through their ability to neutralize free radicals, as mentioned earlier.

### 3.6. Proximate and Texture Profile Analysis after Roasting

The results of a proximate analysis of pork meat patties treated with choline chloride-based DES extracts are presented in Table 4. In the control group, the pH value of meat pie was 6.27, the cooking loss rate was 30.8%, the protein content was 13.2 g/100 g, the moisture content was 56.7%, and the ash content was 1.39%. These values were found to be comparable with those reported in the literature. Within each group, the pH values, protein content, moisture, and ash contents displayed similarity and exhibited a non-significant alteration (*p* > 0.05) when comparing the control group with the patties treated with citrus peel extract. However, patties treated with choline chloride-based DES extracts showed a significant effect on the cooking loss of pork meat patties, which varied from 23.5% to 26.1% and were significantly (*p* < 0.05) lower than that of the control patties.

Table 4 presents the findings from a textural profile analysis of grilled pork meat patties. The pork meat patties treated with DES showed a significant change (*p* > 0.05) in texture profile compared to those without treatment. These changes were observed in hardness, cohesiveness, gumminess, and chewiness. In particular, DES3 displayed significantly lower values (*p* < 0.05) for hardness, cohesiveness, gumminess, and chewiness when compared to both the control and other DES groups. These results may be attributed to the reduced cooking loss in pork meat patties resulting from the addition of DES.

### 3.7. UHPLC–ESI-QTOF–MS Analysis of Citrus Peel Extract

**Figure 3:**
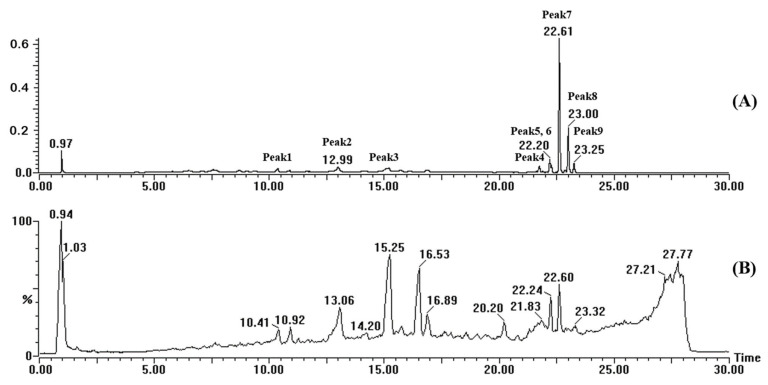
The UV chromatogram (**A**) and total peak ion (**B**) and of citrus peels extract.

Previous studies have shown that PMFs are abundantly present in *Citrus* species, particularly in the peels of sweet oranges (*Citrus sinensis* L. *Osbeck*) [20]. Considering the excellent antioxidant activity and inhibitory effects on HAs and AGEs in grilled pork meat patties demonstrated by citrus peel extract, this study further identified the main active components in citrus peel extract. An UPLC-QTOF-MS/MS system in positive ion mode was used to analyze citrus peel extracts, which were based on choline chloride DES. The purpose was to definitively confirm the presence of flavonoids and other phenolic compounds extracted from citrus peels. The total peak ion and UV chromatogram of citrus peels extract are shown in Figure 3. The comparison of their retention times and MS data with previously reported data in the literature is shown in Table 5 [22,23,39]. Furthermore, the results were confirmed using the chemSpider database and phenol-Explorer database. When reference standards were accessible, the identification of flavonoids and phenolic compounds involved comparing their retention time, UV spectra, MS spectrum with those of the standards. A total of three phenolic compounds, Vicenin, Diosmetin 6,8-di-C-glu, Limonin-hex, and six PMFs, Hesperidin, Isosinensetin, Sinensetin, Tetramethoxyflavone, Tangeretin, and Hexamethoxyflavone, were found in citrus peel extracts.

## 4. Conclusions

Present results indicate that choline chloride-based DES extracts of citrus peel are an effective way to inhibit the production of both free and protein-bound HAs and AGEs during roasting. The physical and chemical changes of citrus peel extracts on the pork meat patties were assessed in the current study. Furthermore, it was determined that the primary reason for the capability of citrus peel extracts to inhibit HAs and AGEs is the suppression of free radicals, lipid, and protein oxidation. Additionally, six kinds of PMFs in citrus peel extract have been identified, which are likely the major active components in the extract.

## Figures and Tables

**Table 1:** The composition of nine kinds of deep eutectic solvents.

Name of DES	Component 1 (HBA)	Component 2 (HBD)	Molar Ratio
DES1	Choline chloride	Acetamide	1:2
DES2	Choline chloride	Ethylene glycol	1:2
DES3	Choline chloride	Carbamide	1:2
DES4	Betaine	Acetamide	1:2
DES5	Betaine	Ethylene glycol	1:2
DES6	Betaine	Carbamide	1:2
DES7	L-carnitine	Acetamide	1:2
DES8	L-carnitine	Ethylene glycol	1:2
DES9	L-carnitine	Carbamide	1:2

**Table 2:** Suppressive effect of citrus peel extract on the production of free and bound heterocyclic amines in grilled pork meat patties. Different lowercase letter denotes significant differences (*p* < 0.05).

Free HAs (ng/g)	Control	DES1	DES2	DES3
PhIP	1.99 ± 0.42 ^a^	0.97 ± 0.04 (51.3%) ^b^	1.01 ± 0.07 (49.2%) ^b^	0.63 ± 0.07 (68.3%) ^c^
MeIQx	1.73 ± 0.28 ^a^	0.91 ± 0.04 (47.4%) ^c^	1.13 ± 0.05 (34.7%) ^b^	0.81 ± 0.03 (53.2%) ^d^
7,8-DiMeIQx	0.88 ± 0.04 ^a^	0.63 ± 0.01 (28.4%) ^b^	0.60 ± 0.01 (31.8%) ^b^	0.48 ± 0.02 (45.4%) ^c^
AαC	0.53 ± 0.02 ^a^	0.12 ± 0.00 (77.4%) ^c^	0.23 ± 0.01 (56.6%) ^b^	0.17 ± 0.00 (67.9%) ^c^
Norharman	5.14 ± 0.75 ^a^	3.01 ± 0.02 (41.4%) ^b^	3.35 ± 0.02 (34.8%) ^b^	3.71 ± 0.04 (27.8%) ^b^
Harman	3.72 ± 0.62 ^a^	3.33 ± 0.01 (10.5%) ^a^	2.98 ± 0.02 (19.9%) ^b^	3.01 ± 0.03 (19.1%) ^b^
Total	14.01	8.97 (35.9%)	9.21 (34.3%)	8.81 (37.1%)
**Bound HAs (ng/g)**	**Control**	**DES1**	**DES2**	**DES3**
PhIP	9.04 ± 0.82 ^a^	4.38 ± 0.07 (51.5%) ^b^	4.70 ± 0.05 (48.0%) ^b^	3.34 ± 0.12 (63.1%) ^c^
1,5,6-TMIP	3.82 ± 0.34 ^a^	3.61 ± 0.09 (5.49%) ^a^	3.74 ± 0.12 (2.09%) ^a^	3.01 ± 0.05 (21.2%) ^b^
AaC	1.91 ± 0.12 ^a^	0.84 ± 0.07 (56.0%) ^b^	0.86 ± 0.01 (54.9%) ^b^	0.64 ± 0.04 (66.5%) ^c^
MeIQx	4.13 ± 0.76 ^a^	2.16 ± 0.07 (47.7%) ^b^	2.33 ± 0.28 (43.6%) ^b^	1.98 ± 0.04 (52.1%) ^c^
Harman	367.4 ± 33.4 ^a^	312.2 ± 2.09 (15.0%) ^b^	335.8 ± 3.71 (8.60%) ^a^	297.5 ± 1.12 (19.0%) ^c^
Norharma	543.6 ± 62.6 ^a^	254.8 ± 0.56 (53.1%) ^b^	269.7 ± 0.49 (50.4%) ^b^	223.2 ± 3.2 (58.9%) ^c^
7,8-DiMeIQx	5.17 ± 0.82 ^a^	3.66 ± 0.42 (29.2%) ^b^	2.12 ± 0.42 (58.9%) ^c^	3.27 ± 0.42 (36.8%) ^b^
MeAaC	38.3 ± 2.74 ^a^	32.7 ± 0.28 (14.6%) ^b^	30.8 ± 0.28 (19.6%) ^c^	33.9 ± 0.28 (11.5%) ^b^
Total	973.4	614.3 (36.9%) ^b^	650.0 (33.2%)	566.8 (41.8%)

**Table 3:** Suppressive effect of citrus peel extract on the formation of AGEs in grilled pork meat patties. Different lowercase letter denotes significant differences (*p* < 0.05).

Free AGEs (μg/g)	Control	DES1	DES2	DES3
CML	59.4 ± 1.95 ^a^	31.1 ± 0.02 (47.6%) ^c^	35.6 ± 0.02 (40.1%) ^b^	29.2 ± 0.04 (50.8%) ^d^
CEL	88.1 ± 3.89 ^a^	51.3 ± 0.01 (41.8%) ^b^	42.1 ± 0.02 (52.2%) ^c^	43.8 ± 0.03 (50.3%) ^c^
Total	147.6	82.4 (44.2%)	77.7 (47.4%)	73.0 (50.5%)
**Bound AGEs (μg/g)**	**Control**	**DES1**	**DES2**	**DES3**
CML	2.51 ^a^	2.08 ± 0.07 (17.1%) ^b^	1.95 ± 0.05 (22.3%) ^b^	1.51 ± 0.12 (39.8%) ^c^
CEL	4.88 ^a^	4.04 ± 0.09 (17.2%) ^b^	4.06 ± 0.12 (16.8%) ^b^	3.39 ± 0.05 (30.5%) ^c^
Total	7.39	6.12 (17.1%)	6.01 (18.7%)	4.90 (33.7%)

**Table 4:** Chemical composition and texture characteristics of the grilled meat patties with different citrus peel extract. Different lowercase letter denotes significant differences (*p* < 0.05).

Group	pH	Protein	Cooking Loss (%)	Ash (%)	Moisture Content (%)
Control	6.27 ± 0.05 ^a^	13.2 ± 0.76 ^a^	30.9 ± 1.09 ^a^	1.39 ± 0.07 ^a^	56.7 ± 1.71 ^a^
DES1	6.23 ± 0.01 ^a^	14.3 ± 0.95 ^a^	23.5 ± 0.56 ^c^	1.51 ± 0.05 ^a^	56.6 ± 1.87 ^a^
DES2	6.24 ± 0.03 ^a^	12.9 ± 0.68 ^a^	26.1 ± 1.09 ^b^	1.64 ± 0.24 ^a^	54.8 ± 1.81 ^a^
DES3	6.30 ± 0.03 ^a^	13.6 ± 1.32 ^a^	23.3 ± 1.13 ^c^	1.45 ± 0.05 ^a^	56.6 ± 1.38 ^a^
**Group**	**Hardness (g)**	**Springiness (mm)**	**Gumminess (g)**	**Cohesiveness (g)**	**Chewiness (g)**
Control	4326 ± 76.53 ^a^	0.90 ± 0.03 ^a^	2617 ± 34.7 ^a^	0.67 ± 0.02 ^a^	2306 ± 122 ^a^
DES1	3107 ± 50.6 ^b^	0.87 ± 0.01 ^a^	2062 ± 96.0 ^b^	0.65 ± 0.31 ^a^	1790 ± 73.1 ^b^
DES2	2776 ± 88.2 ^c^	0.92 ± 0.06 ^a^	1764 ± 35.6 ^c^	0.63 ± 0.02 ^a^	1553 ± 37.8 ^c^
DES3	2295 ± 143 ^d^	0.89 ± 0.06 ^a^	1271 ± 148 ^d^	0.57 ± 0.05 ^b^	1107 ± 44.4 ^d^

**Table 5:** Characterization of phenolic and PMFs in citrus peel extracts of by UPLC-Q-TOF-MS/MS.

Peak	R_t_	M^+^	Fragment Ions	Molecular Formula	Compounds	CAS
	(min)	(*m*/*z*)	(*m*/*z*)			
1	10.14	593.1488	353.0683, 383.0770, 297.0777	C_27_H_30_O_15_	Vicenin Ⅱ	23666-13-9
2	12.99	623.1613	383.0778, 413.0846	C_28_H_32_O_16_	Diosmetin 6,8-di-C-glu	98813-28-6
3	14.97	649.2480	443.2060, 605.2650	C_32_H_42_O_14_	Limonin-hex	1180-71-8
4	21.80	609.1819	301.0763	C_28_H_34_O_15_	Hesperidin	520-26-3
5	22.20	373.1291	343.0820, 315.0859	C_20_H_20_O_7_	Isosinensetin	17290-70-9
6	22.38	373.1289	329.0911, 312.0885	C_20_H_20_O_7_	Sinensetin	2306-27-6
7	22.61	343.1082	282.0779	C_19_H_18_O_6_	Tetramethoxyflavone	3162-43-4
8	23.00	373.1187	343.0740, 358.1048	C_20_H_20_O_7_	Tangeretin	481-53-8
9	23.25	403.1299	373.0823	C_21_H_22_O_8_	Hexamethoxyflavone	478-01-3

## Data Availability

Data is contained within the article.
